# Far from the Familiar: My First Encounter with Chagas

**DOI:** 10.4269/ajtmh.25-0234

**Published:** 2025-07-10

**Authors:** Ozioma Esther Onuselogu

**Affiliations:** Eastern Illinois University, Charleston, Illinois, USA

The raccoon lay motionless on the stainless-steel table, its body limp, eyes closed, fur matted from days in the cold. I stood there, scalpel in gloved hand, heart pounding. This wasn’t what I imagined when I left Nigeria to pursue public health in the United States.

Back home, I was no stranger to neglected tropical diseases (NTDs) like schistosomiasis, lymphatic filariasis, and other tropical diseases such as malaria. These weren’t abstract terms in textbooks; they were real, relentless threats woven into the rhythm of life. In my childhood community, people lived with these diseases, suffered their consequences, and often lacked the resources to fight back. It was the pain and persistence of these conditions that first ignited my desire to study public health. I had envisioned a future in which I would return home, equipped with knowledge to help dismantle the injustices of unequal disease burdens.

But fate had other plans, leading me far from the familiar struggles of my homeland to a cold parasitology lab, where a lifeless raccoon lay before me.

The turn in life that brought me to this racoon began one evening during a graduate parasitology lecture, when my professor introduced us to a parasite I had never heard of: *Trypanosoma cruzi*, the parasite responsible for Chagas disease. My ears perked up. A neglected tropical disease… in the Americas? I listened closely as he described a silent, slow killer, endemic to Latin America but increasingly found in parts of the United States. It was through the research led by Vandermark and my professor, Dr. Elliott Zieman in Illinois, that I first understood how wildlife could hold clues to the spread of this disease.

Transmitted by kissing bugs, this parasite silently invades the body and hides in tissues. Over the course of many years, it gradually damages the heart, often without symptoms until the disease has progressed too far. I was stunned. How could something so dangerous be entirely absent from my worldview, especially as someone who grew up surrounded by NTDs? That night, I fell down a rabbit hole of research. I read about the parasite’s transmission cycles, its insect vectors, the ecological niche it occupies, and the invisibility of the disease in public discourse. It was both interesting and infuriating. Chagas was different from diseases I knew. Though neglected, silent, and deadly, it existed in a context I had never imagined.

I couldn’t shake it. Within days, I approached my thesis advisor, a seasoned parasitologist. “I want to study *T. cruzi*,” I said. “I need to understand how something this serious can go so unnoticed.” My advisor didn’t hesitate. “Then let’s get to work.”

And so began a journey I never anticipated—one that would not take me to mosquito nets and blood slides, but to roadkill, wildlife reservoirs, and DNA extraction kits.

A few days later, I was back in the lab, this time face-to-face with my first raccoon. My advisor, a calm and methodical parasitologist, guided me through the steps of necropsy. Still, I hesitated. I had never worked on animal dissections, let alone with roadkill. My hands trembled slightly as I made the first incision. The smell, the texture, the intimacy of cutting into a creature that once lived, was all new, jarring, and oddly sacred.

As we extracted tissues, heart, spleen, and muscle for DNA analysis, my thoughts raced. Somewhere in these samples might be a clue to an invisible epidemic. The idea that this animal, found just miles from rural and urban neighborhoods, could harbor a parasite capable of causing deadly cardiac disease was both sobering and invigorating.

Weeks passed, and I immersed myself in molecular biology: DNA extractions, PCR, and gel electrophoreses. My days were filled with loading samples, calibrating machines, and squinting at faint gel bands under UV light. Some days, the results were smudged or inconclusive. Other days, there was nothing at all, only blank lanes that mocked my effort. I repeated experiments, tweaked protocols, and checked reagents again and again. I was chasing something I could not yet see but refused to believe wasn’t there.

Then, one late evening, it happened.

Under the ultraviolet glow of the electrophoresis machine, a crisp, bright band appeared. Positive for *T. cruzi* DNA. My breath caught in my throat. After weeks of uncertainty, failure, and doubt, the parasite had revealed itself. I had found evidence of Chagas disease in a raccoon right here in the U.S., thousands of miles from the tropics. It was a strange moment of triumph: exhilarating, humbling, and deeply grounding. In that instant, I was more than a graduate student, I became a witness to the hidden story of a disease most people never even heard about.

That night, I walked home with new clarity. My work wasn’t just about one parasite or one host species. It was about drawing attention to a blind spot in global health. I found myself asking, “Why am I, a Nigerian public health student, studying a disease that doesn’t affect my country”? That question lingered, but the answer soon became clear because diseases don’t recognize borders. They don’t need passports. They don’t care if you are in Nigeria or New Orleans. They move silently, adapting, evolving, and waiting to emerge. Neglect anywhere is a threat everywhere. Chagas disease reminded me not only of the fragility of life, but also of the hidden narratives embedded in our environments, stories that remain silent until illness brings them to the surface. That raccoon, once just a whimsical image on a T-shirt, became a lasting symbol of how personal and global health journeys often intersect in the most unexpected ways.

That realization changed my perspective. Global health, I now understood, isn’t about geography, it’s about interconnectedness. The same ecological pressures that drive malaria in Nigeria can foster Chagas disease in the Americas. Our planet is one ecosystem. When we ignore the silent spread of pathogens in one part of the world, we invite consequences in another.

That raccoon on the table taught me more than any textbook. It taught me that neglect itself is the most dangerous pathogen. It reminded me that the pursuit of public health is not always tidy or linear, sometimes it begins with confusion, curiosity, and a willingness to explore the unknown.

My journey began with a lecture and a leap of curiosity. It led me into a lab, into roadkill, into genetic code, and into the heart of a parasite that changed the way I see the world. It is a story of stepping far outside what I knew, and finding in the process, a purpose more profound than ever imagined.

## Supplemental Materials

10.4269/ajtmh.25-0234 Supplemental Materials

