# Supplementary figures and images for: Far from the Familiar: My First Encounter with Chagas
Source: Am J Trop Med Hyg. 2025 Jul 10;113(3):490–1. doi: 10.4269/ajtmh.25-0234 (PMC12410261; doi:10.4269/ajtmh.25-0234)

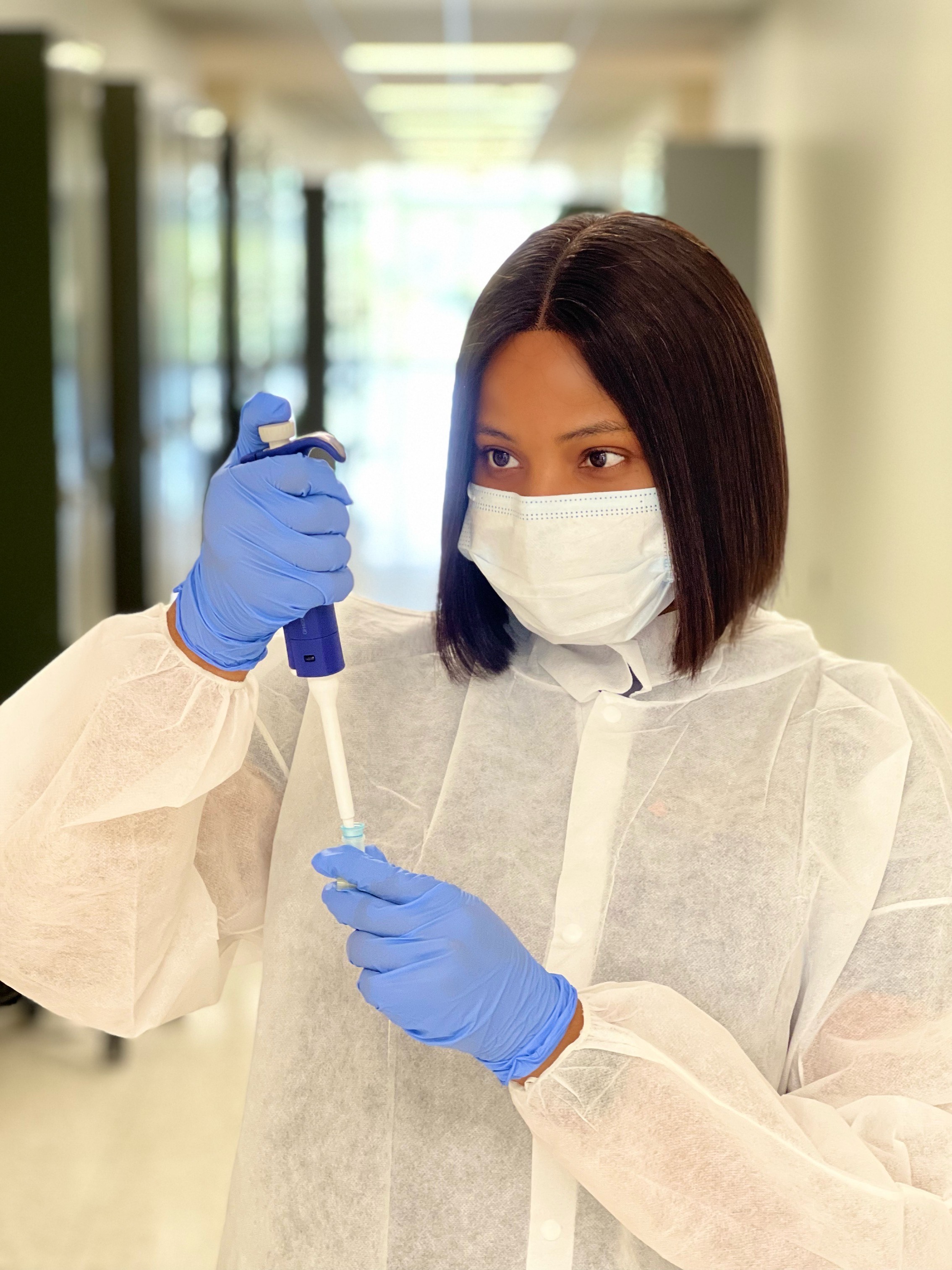

Supplement: Supplemental Materials [file tpmd250234.SD1.jpeg]

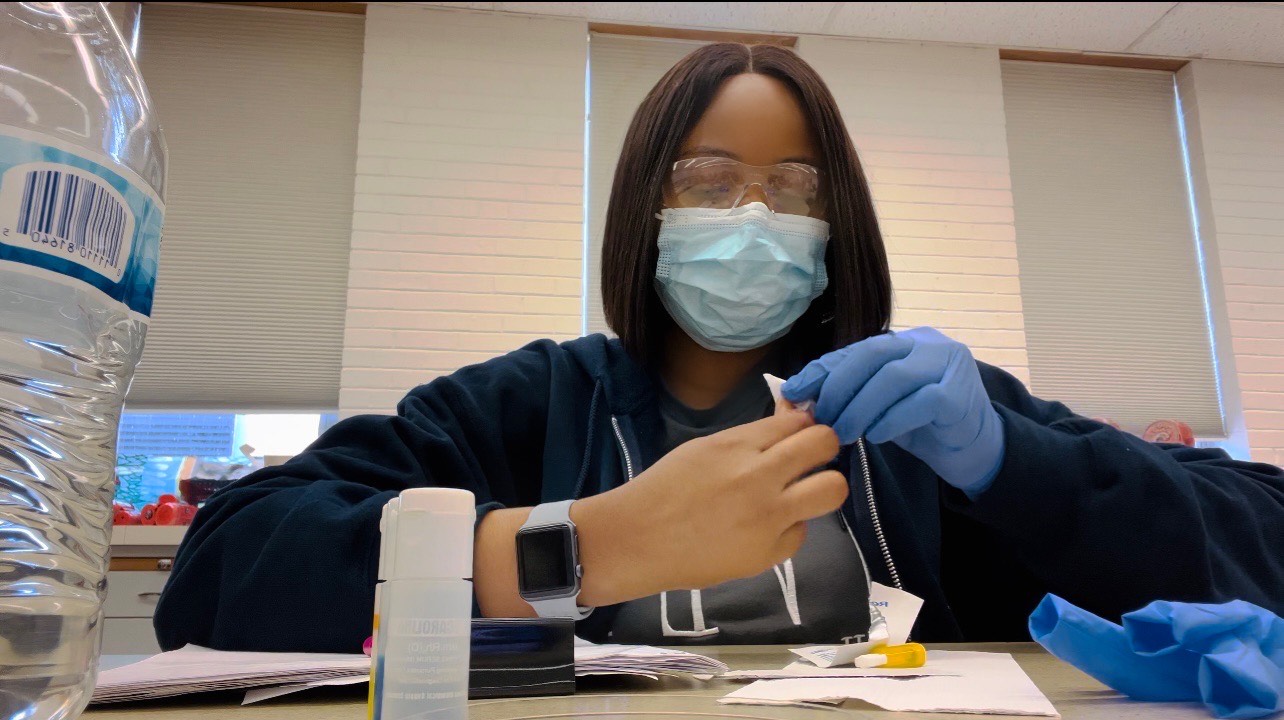

Supplement: Supplemental Materials [file tpmd250234.SD2.jpeg]

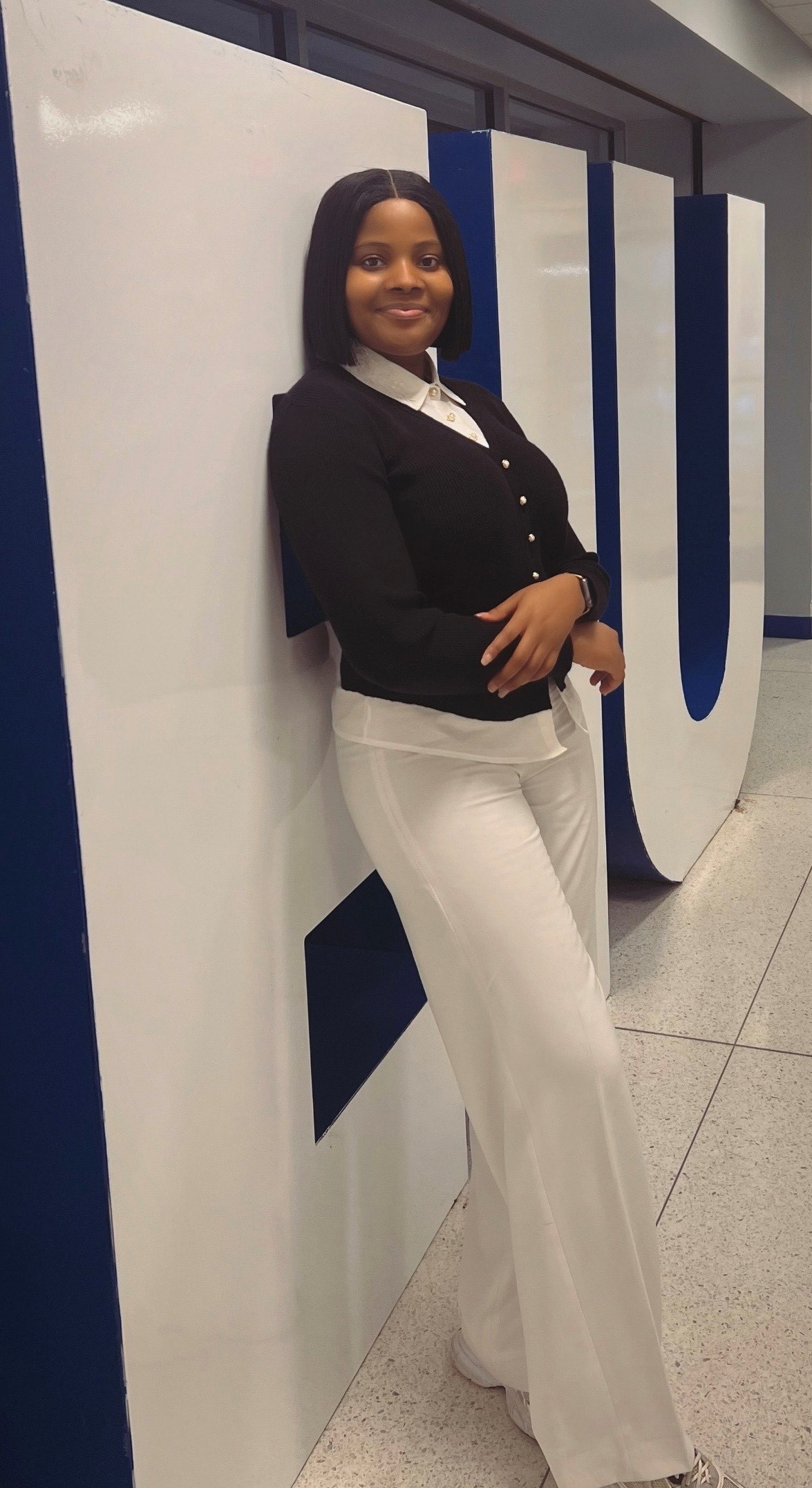

Supplement: Supplemental Materials [file tpmd250234.SD3.jpg]
